# Full Field Masking Causes Reversals in Perceived Event Order

**DOI:** 10.3389/fnins.2020.00217

**Published:** 2020-03-17

**Authors:** Samson Chota, Douglas McLelland, Louisa Lavergne, Eckart Zimmermann, Patrick Cavanagh, Rufin VanRullen

**Affiliations:** ^1^CerCo, Université de Toulouse Paul Sabatier, CNRS, Toulouse, France; ^2^Université de Paris, Laboratoire Vision Action Cognition EA7326, Paris, France; ^3^Faculty of Mathematics and Natural Sciences, Institute for Experimental Psychology, Heinrich Heine University Düsseldorf, Düsseldorf, Germany

**Keywords:** temporal order judgements, saccadic compression, temporal reversal, masking, temporal distortion, peri-saccadic illusion

## Abstract

We generally experience a stable visual world in spite of regular disruptions caused by our own movements (saccades, blinks) or by the visual input itself (flashes, occlusions). In trying to understand the mechanisms responsible for this stability, saccades have been particularly well-studied, and a number of peri-saccadic perceptual distortions (spatial and temporal compression, failure to detect target displacement) have been explored. It has been shown that some of these distortions are not saccade specific, but also arise when the visual input is instead abruptly and briefly masked. Here, we demonstrate that another peri-saccadic distortion, the reversal of the temporal order of a pair of brief events, may also be found with masking. Human participants performed a temporal order judgment task, and the timing of stimuli and mask was varied over trials. Perceptual order was reversed on ~25% of the trials at the shortest stimulus to mask intervals. This was not merely a failure of target detection, since participants often reported these reversals with high subjective confidence. These findings update the constraints on models of stability around disruptions.

## Introduction

Our visual system is continually challenged by disruptions, both in the form of externally imposed interruptions and internally generated saccadic eye movements. Characterizing the way that our brains build perceptual continuity in the face of these events can yield useful insights into the underlying mechanisms, as can the errors that are generated in the process.

Saccadic eye movements have been particularly well-studied in this regard, with several peri-saccadic illusory percepts characterized: displacement of an object during a saccade may go unnoticed [“saccadic suppression of displacement,” (Bridgeman et al., 1975; Deubel et al., 1996)] and both the spatial and temporal separation between briefly presented objects around saccade time is compressed (Ross et al., 1997; Lappe et al., 2000; Morrone et al., 2005). These features were long identified with saccades and so were principally discussed within that context (Melcher and Colby, 2008). However, a number of studies have suggested that similar effects can be obtained during fixation if the visual scene is disrupted, by a saccade-mimicking shift of the stimuli (Mackay, 1970; O'Regan, 1984; Ostendorf et al., 2006), or even by simpler manipulations such as flicker (Terao et al., 2008) or a brief visual mask (Zimmermann et al., 2014). This has led to the suggestions that these illusory percepts are not limited to saccades, but that the spatial and temporal compression may reflect more general mechanisms responsible for visual continuity in the face of disruptions (Zimmermann et al., 2014).

In the course of working with the masking paradigm used by Zimmermann et al. (2014), we noted another perceptual illusion: temporal reversal of a pair of brief visual events, that is, the later of two stimuli was perceived as occurring first. Again, a comparable illusory percept has previously been described for saccades (Morrone et al., 2005). To pilot our observation, we presented the demonstration video (Supplementary Video 1) as a repeated loop to 19 colleagues and found that 13 of them (including 4 out of 6 authors) reported seeing a temporal reversal (that is, they perceived the yellow probe as occurring before the red target).

During our pilot, the reports of mask-triggered, perceptual reversals were variable and did not reliably occur in every participant. When tested in the context of saccades, Morrone et al. (2005) reported a maximum reversal rate of almost 100% for their two participants as did Binda et al. (2009); however, further reports, also with saccades, suggest much lower probability of reversal perception on average (Kitazawa et al., 2007; Kresevic et al., 2016). In particular, Kresevic et al. (2016) found that just 7 of their 11 participants experienced robust occurrence of reversals with substantial interindividual variability in the proportion of reversals and the timing of the peak effect.

Here, we describe and formally test a masking procedure that yields this illusory reversed order percept in the majority (9 out of 14), but not all participants, and present results characterizing the temporal dynamics of the illusion (the effects of relative timing between the successive visual targets and the mask).

## Materials and Methods

### Participants

Fourteen participants (8 male) were tested, aged between 21 and 34 years old. All were naive to the goals of the experiment. All participants provided written informed consent. They were not preselected based on perception of the reversal in the demonstration video, and no authors were included. The experiment was carried out in accordance with the protocol approved by the Centre National de la Recherche Scientifique ethical committee and followed the Code of Ethics of the World Medical Association (Declaration of Helsinki).

### Stimulus

**Figure 1:**
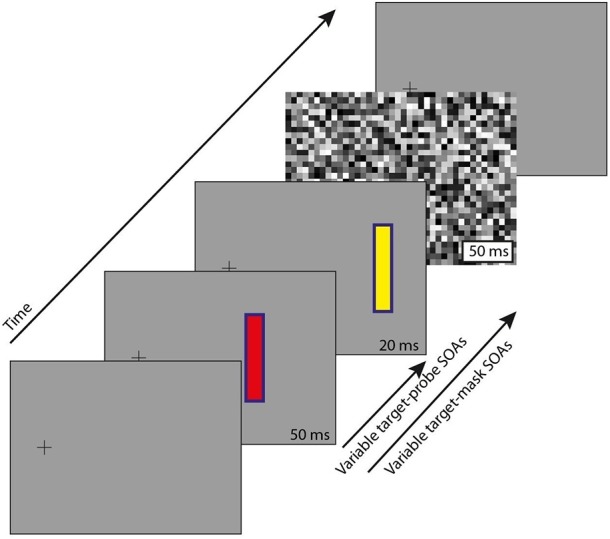
Stimulus paradigm. Participants maintained fixation on the black cross throughout. After a variable delay from trial start (300–800 ms), a target appeared (red, 50 ms duration), and with variable SOA around that (−20 to 160 ms) a probe (yellow, 20 ms duration). Note that the target and probe are at non-overlapping locations (see text for details). A full-field pixelated mask was presented (50 ms duration) at one of 3 delays after target onset: 50, 100, or 150 ms. There was also a no-mask control condition. For a demonstration of the illusion we refer to the video in the Supplementary Material.

The stimuli (Figure 1) comprised a fixation point, two briefly flashed objects at variable stimulus onset asynchronies (SOAs), and a full field mask, details as follows.

Stimuli were generated using the Psychtoolbox extension (Brainard, 1997; Pelli, 1997; Kleiner et al., 2007) for Matlab (Mathworks), and presented on a CRT monitor (100 Hz frame rate, 800 × 600 pixel resolution, 36.5 × 27.2 cm screen) at a distance of 57 cm from participants, who were seated with head position maintained at the center of the monitor by a chinrest. Stimuli were presented against a mid-gray background (CIE xyY 0.30 0.30 6.1). The fixation point was a black cross, situated toward the left of the screen (7.2° of visual angle from midline), at mid height, and was present continuously. The eccentric position of the fixation cross served to simulate conditions for an experiment with saccades toward the targets.

Two stimulus objects were presented to the right of the fixation point, also at mid screen height. The first, which we shall refer to as the “target,” appeared with unpredictable timing relative to trial start (equal probability from 300 to 800 ms), for a duration of 50 ms. All other timings are described relative to target appearance. The target was presented 7.2° to the right of the fixation point, and comprised a red rectangle (CIE 0.54 0.32 4.65) with a 2-pixel wide blue border (CIE 0.18 0.15 1.8), the whole measuring 3° tall by 0.9° wide.

The second stimulus object, which we shall refer to as the “probe,” was presented 2° to the right of the target, and with similar characteristics, except that the rectangle fill was yellow (CIE 0.41 0.48 11.6). The probe was presented for a duration of 20 ms, with variable SOA relative to the target across trials, from 20 ms before to 160 ms after, in 10 ms steps. The choice of SOA's and stimulus duration was determined by prior piloting to maximize illusory reversals.

The mask comprised a full-screen flash of pixelated white noise (see Figure 1), with square “pixels” measuring 1.1° each side, and each pixel taking a random luminance value (equal probability from minimum screen luminance, CIE 0.13 0.08 0.0, to maximum screen luminance, CIE 0.29 0.30 17.1). The mask was presented for a duration of 50 ms, with variable SOA relative to the target across trials, at 50, 100, or 150 ms. When mask and probe were simultaneously present, the probe was always drawn over the mask (that is, the mask did not directly conceal the probe).

There was also a control condition in which no mask was included. In total, 76 conditions (19 probe SOAs × [3 mask SOAs + 1 control]) were presented in random order, with 25 repeats of these blocks resulting in 1,900 trials per subject. All trials were collected over the course of 1–2 sessions per participant.

The participants' task on each trial was to report whether the target (red/leftmost) or probe (yellow/rightmost) appeared first, using a key press (target, “F”; probe “J”). If unsure, participants could indicate this (while still making a forced target/probe first choice) by using a different key pair (target, “G”; probe, “H”). Responses were not time limited, and the response key press launched the next trial. Pauses were included between blocks (terminated by the participant pressing the space key).

To enable readers to experience the illusion for themselves, we include a video of the stimulus paradigm (Supplementary Video 1), with timings that typically generate the illusion on a proportion of trials in most participants using a typical monitor with 60 Hz refresh rate: target duration 50 ms, target:probe SOA 117 ms, probe duration 17 ms, target:mask SOA of 150 ms, mask duration 50 ms, although note that the precise timings obtained will obviously depend on the video display equipment used.

### Data Analysis

Data were analyzed and prepared for presentation using Matlab (Mathworks). For single participant performance data, we show the maximum likelihood estimate (*mle*) with 95% confidence intervals (*CI*; Clopper-Pearson method, Matlab *binofit* function). Where the difference from control performance is shown, we again show *mle* with 95% *CI* [Newcombe's CI, see (Newcombe, 1998; Brown and Li, 2005)]. For the group averages, we show the mean ± standard error (*SEM*; *n* = 14 throughout). Maximum likelihood estimates were calculated for each participant individually and then averaged to obtain mean and standard errors displayed in the group averages.

Statistical analysis of the group averages was calculated by performing *t*-tests for each Target:Probe SOA. *P*-values were corrected for multiple comparisons using False Discovery Rate (fdr). Statistical analyses of single participant data was performed using a Chi-square test with Yates correction. For single participant statistical analysis, only the peak SOA's at 10 and 20 ms prior to mask (selected based on the group average) were included.

## Results

**Figure 2:**
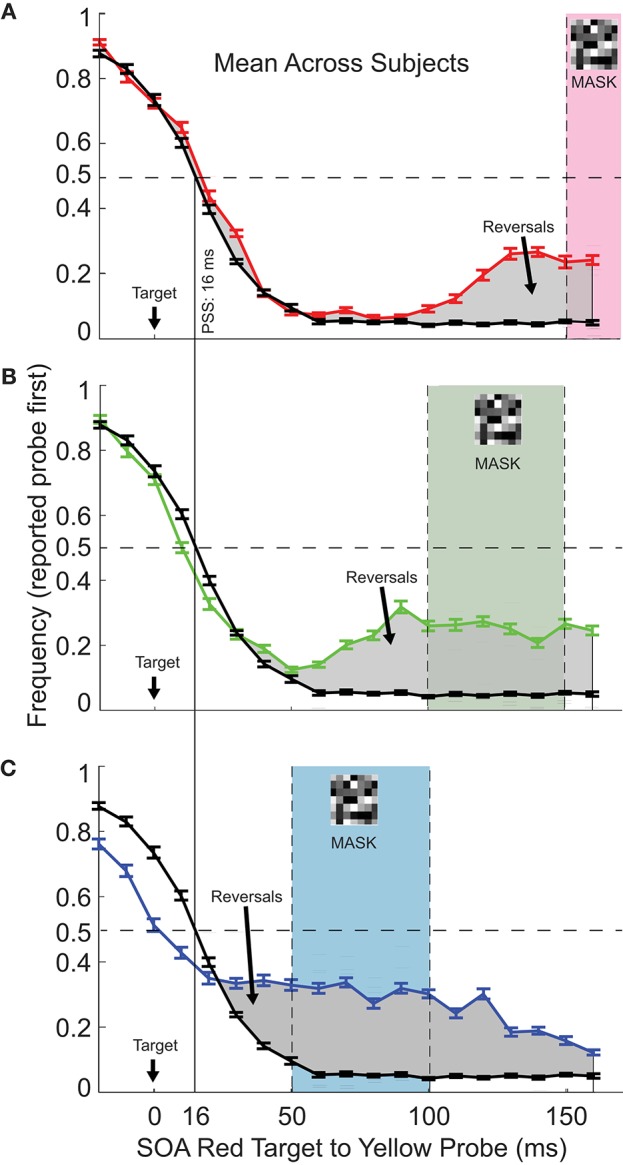
The frequency of reporting probe first as a function of Target:Probe SOA and mask timing. **(A)** The red curve shows mean performance (*N* = 14) for mask onset at 150 ms after the red target onset (mask timing indicated by the shaded area on the plot). The black curve, this plot and the following plots, shows performance in the control task, with no mask included. The vertical line indicates the Point of Subjective Simultaneity (PSS). For most probe timings, performance was unaffected by the inclusion of the mask. However, when probe onset was close to the time of mask onset, performance fell, that is, on a substantial fraction of trials, the probe was reported as occurring before the target, in spite of a >100 ms delay between them. Gray shaded areas indicate SOA's for which reversals were observed. **(B,C)** As for **(A)**, except that the mask onset was at 100 ms **(B)** or 150 ms **(C)**. Note that the same control curve is duplicated across plots for ease of reference. Error bars show ±1.0 *SEM*.

For the control task with no mask (black curves repeated in each of Figures 2A–C), the psychometric function followed a predictable trend, with accurate reporting of “probe first” (yellow first) for early target-probe SOAs, and “target first” (red first) for late (>95% accuracy for SOAs of <0 and >50 ms). Bearing in mind that the red target (50 ms duration) had a longer presentation time than the yellow probe (20 ms), the expected point of subjective simultaneity is at an SOA of 15 ms, assuming that perceptual timing for these brief stimuli is based on the midpoint of stimulus duration. This assumption appears to hold up as the data show a value of about 16 ms for subjective simultaneity (Figure 2A).

Figure 2A (red trace) shows the effect of including the mask 150 ms after the red target onset. For most target-to-probe SOAs, performance was unchanged from the control condition (black trace). However, when the mask closely followed the yellow probe (probe to mask intervals of 20 ms or less, SOA of 130 ms or more), the probe was erroneously perceived as occurring before the target on a about 25% of the trials, in spite of the long target to probe delay. We will refer to these perceptual errors as “temporal reversals.”

Figure 2B (green trace) shows that effects were similar for mask 100 ms after the red target onset, with temporal reversals observed for probe timing close to or during the mask, reaching over 30% reversals for the probe to mask interval of 10 ms.

Reversals were again observed for mask onset at 50 ms following the red target offset (Figure 2C). In this condition, it is clear that the reversals continue after the mask presentation. Note also that this early mask condition showed some reversals even when the yellow probe physically preceded the red target (the two leftmost tests at negative SOA in Figure 2B). In this case, the yellow probe was erroneously reported as following the red on about 10% of the trials.

Importantly the mask did not have to temporally overlap with the probe in order to induce reversals: they could arise for probe onsets 30 ms or more before the mask onset (Figure 2B; again, note that probe duration was 20 ms) or after mask offset (Figure 2C).

**Figure 3:**
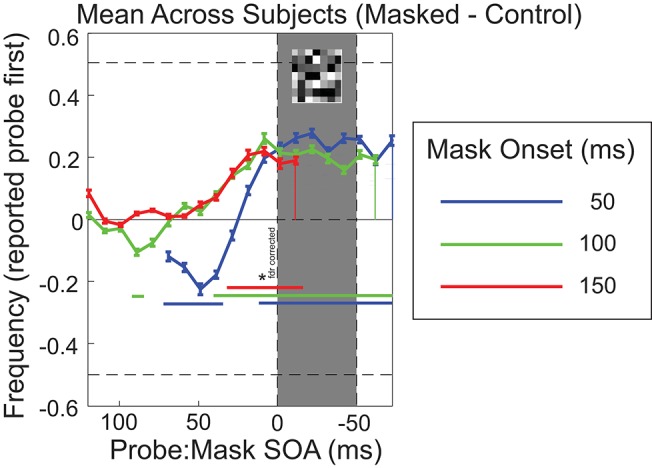
Task performance aligned to mask onset, showing difference from control (no mask) performance (masked minus control). Mean result across participants (*n* = 14; error bars show *SEM*). Blue curves show results for mask onset at 50 ms, with mask timing indicated by the shaded gray area; likewise, green curves for mask onset at 100 ms, and red curves for mask onset at 150 ms. Blue, green and red horizontal bars denote the SOA's for which significantly more reversals were reported compared to 0 (students *t*-test, *p* < 0.05, false discovery rate correction).

Results compared to the control baseline with the data aligned to the mask onset are shown in Figure 3. *T*-tests on the group averages revealed several SOA's for which significantly more reversals were reported in all three mask conditions. Especially when probes were presented immediately prior to mask, reversals are frequently reported (students *t*-test, *p* < 0.05).

### Individual Differences

**Figure 4:**
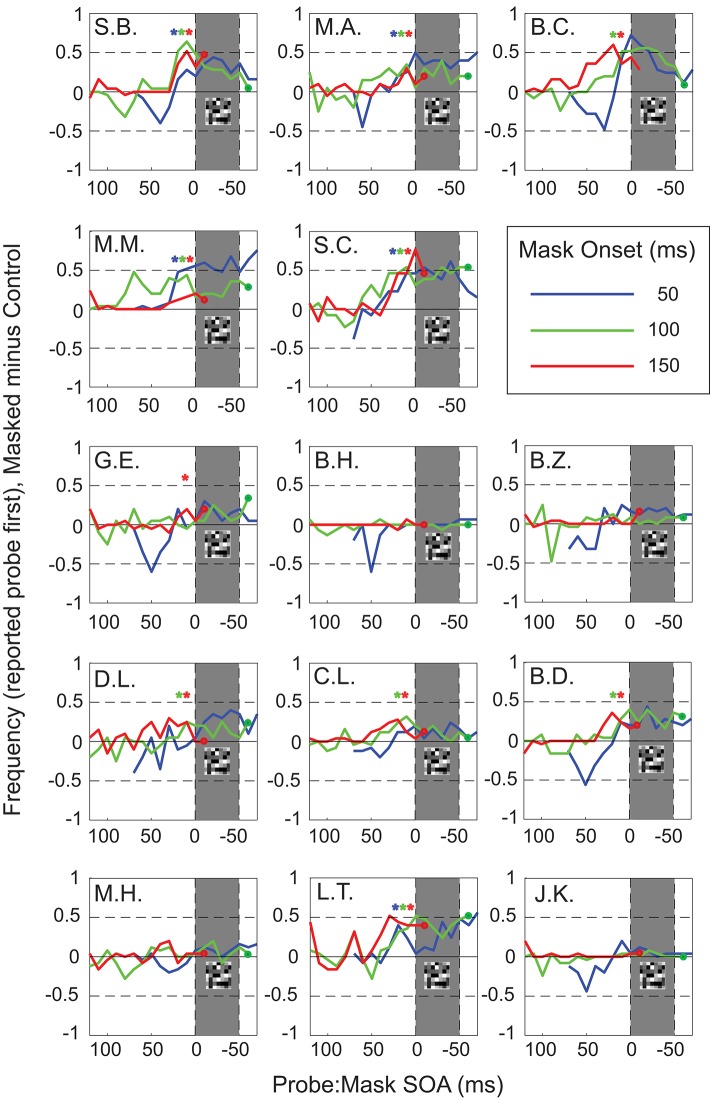
Task performance aligned to mask onset (masked minus control). Results for individual participants (mle). Blue curves show results for mask onset at 50 ms, with mask timing indicated by the shaded gray area; likewise, green curves for mask onset at 100 ms, and red curves for mask onset at 150 ms. Colored stars indicate significant increases in reported reversals compared to 0 (control/no mask) for blue (50 ms), green (100 ms), and red (150 ms) mask conditions at 10–20 ms before mask onset.

The individual results were quite variable. Five participants (Figure 4, S.B., M.A., M.M., S.C., L.T.) showed significant effects in all three masking conditions, with strong temporal reversals around mask timing for positive target:probe SOAs (Chi-square (with Yates correction, *p* < 0.05) tested on SOA 10–20 ms before mask onset). Four more participants (Figure 4, B.C., D.L., C.L., B.D.) showed a significant number of reversals in 2 out of 3 masking conditions. The remaining subjects showed significant reversals only in one (G.E.) or in none of the conditions (M.H., B.Z., J.K., B.H.).

We also observed some reversals for negative SOAs in the early mask condition in 10 out of 14 subjects. This was not unexpected since negative Probe:Mask SOA's in the early mask condition mimic the experimental parameters of positive Probe:Mask SOA's by simply swapping the timing of Probe and Target relative to the mask.

**Figure 5:**
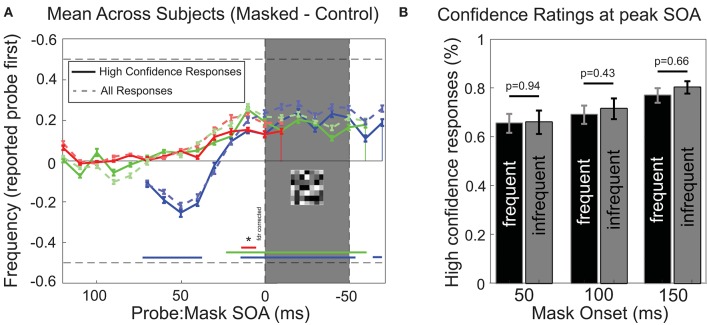
Analysis of confidence ratings. **(A)** Performance for high-confidence only responses. As Figure 3, except that only trials where participants responded with high confidence were included (mean across participants, ± *SEM*; *n* = 14). Faint dashed traces in the background show results for all trials for comparison. **(B)** Confidence ratings for the two SOA's for which reversals were reported most frequently [10 and 20 ms before MASK onset, see **(A)** for reference]. Participants were statistically divided into two equal groups, one “frequent illusion” (black bars) group and one “infrequent illusion” group (gray bars), based on the number of reversals that were observed for each subject for SOA's 10–20 ms before Mask [Chi-square test with Yates correction (*p* < 0.005)]. No significant difference in confidence was observed between subjects reporting frequent reversals and subjects reporting infrequent reversals for any mask condition (students *t*-test, *p* < 0.05). Error bars represent SEM.

Participants were also required to include an indication of “high” or “low” confidence in their response on each trial (see Methods). High confidence trials indicate that participants clearly perceived the relative timing and detected both stimuli. Figure 5A shows that the analysis of only high confidence trials (78.7% of trials) resulted in a very similar pattern of temporal reversals over time, and only a slight reduction in the proportion of reversals reported. This shows that on trials where participants reported reversals, they most often had a clear subjective perception of both stimuli and their (illusory reversed) temporal order. Figure 5B shows the confidence ratings reported at the SOA's for which the strongest effects were observed (10–20 ms before mask, for every mask condition, respectively). We separated subjects into two equal groups, one “frequent illusion” group, showing strong perceptual reversals and one “infrequent illusion” group, showing weak perceptual reversals. Separation was based on the frequency of reversals observed for SOA's prior to mask [10–20 ms, determined by a Chi-square test (*p* < 0.005)]. We did not find any significant differences in confidence ratings between the frequent reversal group (S.B., M.A., B.C., M.M., S.C., C.L., L.T.) and the complementary infrequent reversal group for any condition (students *t*-test) suggesting that differences in reported confidence do not suffice to explain perceived reversals.

## Discussion

In this study, we have described a visual illusion whereby the temporal order of a pair of stimuli may be perceptually reversed when a visual disruption (full field mask) is presented.

This illusion resembles the phenomenon of temporal reversal previously described for events close to the time of saccades (Morrone et al., 2005; Kresevic et al., 2016). It has recently been shown that other visual phenomena, typically considered as “peri-saccadic,” can also be produced by visual masking in the absence of saccades: spatial compression, temporal compression, and suppression of displacement (Zimmermann et al., 2014). Those results were taken to imply that the mechanisms underlying these phenomena might not be saccade specific, but rather reflect a more general mechanism to match corresponding visual objects across disruptions in space and time. Of course, despite the similarities between saccade- and mask-induced phenomena, the possibility remains that different or additional mechanisms are at play in the case of saccades. The present results contribute to this discussion as they show that yet another peri-saccadic phenomenon (temporal reversal) occurs for externally imposed visual disruptions.

While the original report of peri-saccadic temporal reversal presented data from a pair of participants who each experienced very robust reversals (approaching 100% of trials for optimal SOAs; Morrone et al., 2005; Binda et al., 2009), more recent reports suggest much lower probability of reversal perception on average (Kitazawa et al., 2007; Kresevic et al., 2016) similar to the levels we found here for mask-driven reversals. In particular, Kresevic et al. (2016) present individual data from 11 participants that emphasize the strong inter-participant variability in the perception of perisaccadic reversals: only 7 of their 11 participants experienced robust occurrence of reversals (greater than chance report level; around 80% reversals for each of those participants). Other participants reported 50% or lower reversals, and substantial variability in the timing of peak effect. The amplitude, time course and inter-participant variability of the effects are comparable for the saccade- and masking-triggered reversals. In particular, in the data of Kresevic et al. (2016), the peak timing for reversals was between 31 and 70 ms before the saccade onset (for 7 of 11 participants, including all of those with greater than chance levels of reversal). Since their two stimuli were separated by 50 ms, their peak effect occurred when the second stimulus appeared in the ±20 ms around saccade onset. This is comparable to our observed probe to mask timing for the peak effect seen in Figure 3 where it is clear that most temporal reversals are perceived when the time of probe presentation is just before or overlaps with the mask.

There are a number of suggestions for the origin of these temporal reversals, and here we will describe proposals arising from saccade and masking studies, as well as address possible confounds. First, it could be suggested that temporal reversals are simply an exaggerated version of temporal compression, that proportion of trials on which temporal compression was extreme enough to reverse the apparent order of stimuli. Both reversals and compression are found perisaccadically (Morrone et al., 2005; Binda et al., 2009) and with masking (Zimmermann et al., 2014). Binda et al. (2009) proposed that temporal compression could account for the perception of reversals in a temporal order. Briefly, the probability of observing a reversal under such a model depends on the overlap between two probability distributions, one each for the perceived timing of the “target” and the “probe.” Reversals can occur within the overlapped regions and as compression pushed the distribution means together, the overlap increased, and according to the authors' model, so did the frequency of reversals. However, those results were for very short interstimulus intervals in the temporal order judgment task (8 or 20 ms) where there is clearly a large overlap between the distributions of the two judgments. This explanation cannot easily be extended to our data, with far longer intervals of up to 160 ms and more. To do so, the mask would have to dramatically broaden the probability distribution for the probe so that it overlapped with that of the much earlier target and, if this were the case, there should be more reversals for the conditions with shorter intervals between the target and the probe where there would be even more overlap. This did not happen: of the 9 participants showing high levels of reversals for late probes (Figure 4). Five of them show higher peak levels of reversals for longer vs. shorter target to probe SOAs (i.e., S.B., green peak higher than blue; S.C., red peak higher than blue).

Second, Kresevic et al. (2016) proposed that their data are consistent with perceptual insensitivity: the neural onset transient for a stimulus near to/during saccade time may be suppressed, leading to temporal ambiguity and necessitating a TOJ inference based on other cues than the unreliable temporal signals. For the majority of participants this would be apparent as a decrease in performance toward chance level, but, depending on biases in the inferential process (e.g., individual differences in assumptions about the temporally ambiguous stimulus, or in the assignment of attention), a subset of participants could show more systematic (substantially above chance) reporting of reversals, a feature apparent in each of the pertinent data sets [current Figure 3; as well as (Morrone et al., 2005; Kresevic et al., 2016)]. A related account is that the visual disruption (mask or saccade) decreases the effective contrast/salience of stimuli, an effect that would be strongest for the stimulus closest in the time to the disruption. It has previously been shown that, in a simple TOJ task, a lower contrast stimulus is often perceived as having occurred before an earlier, higher contrast stimulus (Bachmann et al., 2004), although their finding of temporal reversal was restricted to short (<50 ms) SOAs. Kresevic et al. (2016) tested whether this might account for temporal reversals in their paradigm by repeating the experiment but replacing the TOJ task with a relative salience judgment. They found saccade-related suppression of salience, with timing matching the generation of reversals, supporting the above hypothesis. However, they actually found that salience suppression was more robust than temporal reversals across participants, and so concluded that additional factors must contribute on a participant-specific basis. Nonetheless, salience suppression remains plausible as a contributing factor in the generation of reversals in our mask-based paradigm.

An additional argument against the contribution of general temporal imprecision is that the results calculated only from the higher confidence trials show the same reversals and timing (Figure 5A). Furthermore, when comparing participants who frequently perceived reversals to those who did not perceive the illusion, confidence ratings did not differ (Figure 5B). These data suggest that while *subjectively* the temporal order of stimuli appeared clear to participants, a subset of them nevertheless consistently reported the wrong order, suggesting an illusion rather than an imprecision. To illustrate this we refer to the demonstration video of the stimulus paradigm in the supplementary materials (Supplementary Video 1). The illusion is best seen with the video set to loop repeatedly. Fixation should be maintained on the cross at the left of the window, with attention focused on the yellow (rightmost) bar. The task is to judge the order in which the red and yellow bars appear: a temporal reversal would be evident if the yellow bar were perceived first. Participants who do report reversals do not report difficulty in perceiving both target and probe (once accustomed to the stimuli and attending to the correct spatial location).

Another factor to consider is the potential role of “prior entry,” whereby an attended stimulus gains a temporal advantage in processing, and can be perceived as occurring before an earlier stimulus (Hikosaka et al., 1993; Spence and Parise, 2010), superficially similar to the temporal reversals described here. However, the absence of temporal advantage for the probe in our control (no mask) task showed no evidence of bias favoring the yellow probe over the red target in our stimuli. There are several other paradigms involving temporal reversals that deserve mention, namely masked priming (Scharlau, 2007), attentional blink (Akyürek et al., 2012; Spalek et al., 2012), and motion-induced blindness (Wu et al., 2009). While having some resemblance to our paradigm we think that all of these examples are fundamentally different from the effects that we observe. In the masked priming paradigm of Scharlau (2007) the temporal order of a prime-mask and a subsequent stimulus is probed. No mask is presented that influences the two TOJ stimuli. In contrast we probe temporal order of two stimuli that are followed by a full field mask which has notable results as can be seen in our findings. Attentional blink phenomena are usually measured using rapid serial visual presentation and can be observed at SOA's of more than 100 ms. We observe reversals for much smaller SOA's between Probe and Target, starting at 30 ms and reaching full effectivity at 50 ms (see Figure 2C). Under very specific conditions motion induced blindness can result in temporal reversal of an inhibited and a flashed object. However, since all of our stimuli are clearly visible and at no point of time under the influence of motion induced inhibition we find it difficult to see clear parallels to our findings.

A last potential confound that we want to address are unintentional eye movements during the experiment. Saccades that are time-locked to the stimuli or mask could have potentially resulted in the pattern that we observed through actual peri-saccadic effects as described in the literature (Morrone et al., 2005). However, eye movements that are triggered by the onset of the stimuli should result in very similar patterns in experimental and control conditions. Instead we find that the mask was crucial to reveal reversal effects. It is conceivable that the mask itself triggered eye movements but it is unlikely that this could give rise to our results for two reasons: (1) the mask was a full field noise mask without features that could draw attention. It is therefore highly unlikely that attention would be drawn enough to elicit frequent and consistent eye movements that would be necessary to result in a saccadic effect. (2) if the mask indeed elicited reliable saccades we would expect the reversals to follow the patterns that were previously observed in the literature (Morrone et al., 2005; Kresevic et al., 2016), namely a peak reversal around 50 ms before saccade onset. Assuming a saccadic reaction time of ~100–200 ms in response to our mask, we should expect the peak reversal to happen around 50–150 ms after mask onset, which we clearly show to not be the case, with our peak reversal happening at 10–20 ms prior to mask.

Reversals were reported even for the longest intervals tested between the first and second stimulus (160 ms). From the shape of the curves in Figures 2, 3, this range could plausibly extend tens of ms beyond this. The original report of perisaccadic reversals also included reversals for grouped data from interstimulus intervals of 76–200 ms (Morrone et al., 2005). These values put the temporal reversal phenomenon into the category of illusions which seem to emphasize the re-writeable nature of conscious perception (Eagleman and Sejnowski, 2000; Scharnowski et al., 2009; Herzog et al., 2016): 160 ms is ample time for the signal from the onset of the first stimulus to be processed by much of the cortex, and yet the subsequent appearance of the second stimulus can still be perceived as occurring first.

In particular, Zimmermann et al. (2014) have suggested that peri-saccadic/masking effects reflect a correspondence process that links targets of interest across interruptions in visual input. We would like to close by proposing a model of the temporal aspects of that process. We suggest that after a visual disruption, the representation of the scene must be updated by linking each item that had been attended prior to the disruption to its post-disruption version. Because this process is serial (at least, for items within a certain spatial scope), items are updated, thus *perceived*, one by one. Because this updating process most likely relies on delayed feedback from higher order areas (Enns and Di Lollo, 2000; Fahrenfort et al., 2007; Binda et al., 2009), visual disruption shortly after the probe might interfere with the updating process of the target but not with the probe. It is possible that this disruption causes the updating of the target to be delayed to later position in the sequential updating process and thus its perception is delayed. We assume that the rate at which attention deals with each item in turn is linked to neuronal oscillations in the theta (4–8 Hz) or alpha (8–12 Hz) frequency bands (VanRullen et al., 2007; Busch and VanRullen, 2010; Landau and Fries, 2012; Fiebelkorn et al., 2013). Directly supporting the link between these lines of argument, we recently described a correlation between the phase of a theta frequency oscillation and the amount of perisaccadic mislocalization observed across trials (McLelland et al., 2016). Different theories of oscillatory processing have in common the idea that discrete items would be processed one by one, *serially*, either across (Fries, 2015) or within (Lisman and Jensen, 2013) the cycles of a slow oscillation. A dependency on the oscillatory phase might also explain why illusory reversals are not experienced in every trial. We speculate here that this serial nature of processing could underlie the finding of temporal reversals.

In conclusion, we have shown that masking can result in a perceptual reversal of the temporal order of a pair of brief events. This is reminiscent of the temporal reversal previously described around the onset of saccades, although we cannot demonstrate here that there is a common underlying mechanism. This mask-based paradigm may nonetheless be very useful in exploring mechanisms of temporal order perception at fast timescales, because it affords greater temporal control and repeatability than equivalent saccadic paradigms.

## Data Availability Statement

The datasets generated for this study are available on request to the corresponding author.

## Ethics Statement

The studies involving human participants were reviewed and approved by Centre National de la Recherche Scientifique ethical committee. The patients/participants provided their written informed consent to participate in this study.

## Author Contributions

PC, DM, LL, and RV conceived and designed the experiment. DM, LL, and SC performed the experiments. DM and SC analyzed the data and wrote the manuscript. RV, PC, LL, and EZ edited the manuscript.

### Conflict of Interest

The authors declare that the research was conducted in the absence of any commercial or financial relationships that could be construed as a potential conflict of interest.

## Supplementary Material

Supplementary Video 1 Demonstration video of the stimulus paradigm. The small black square to the left should be fixated. The task is to judge the order of presentation of the red “target” and yellow “probe” rectangles. Best results are obtained when the video is set to loop repeatedly. Over half of the observers tested, formally and informally report seeing the yellow probe first with these parameters. Many also note (although this was not tested) that the two colored bars appear closer together than they actually are as reported previously by Zimmermann et al. (2014).
